# Potential clinical value of circulating tumor cells in predicting progression for atypical teratoid rhabdoid tumor in young children

**DOI:** 10.1007/s11060-025-05293-6

**Published:** 2025-10-28

**Authors:** Wei Zhang, Ke Cao, Xiaoling Zhang, Nianhua Cao, Lidan Xiao, Zongbin Liu, Xiuli Yuan, Jingsheng Wang

**Affiliations:** 1 Department of Neurosurgery, Shenzhen Children’s Hospital, 7019 Yitian Road, Shenzhen, 518038 China; 2 Department of Laboratory, Shenzhen Children’s Hospital, 7019 Yitian Road, Shenzhen, 518038 China; 3 Department of Hematology & Oncology, Shenzhen Children’s Hospital, 7019 Yitian Road, Shenzhen, 518038 China; 4 Shenzhen Zigzag Biotechnology Co., Ltd, Shenzhen, 518107 China

**Keywords:** Circulating tumor cells, ATRT, Liquid biopsy, Pediatric brain tumor

## Abstract

**Objective:**

Atypical teratoid rhabdoid tumor (ATRT) is a rare pediatric brain tumor characterized by an extremely poor prognosis despite receiving comprehensive treatments. Circulating tumor cells (CTCs) detection has high clinical value in the prediction of the progression of malignant solid tumors and the evaluation of treatment effects. However, very few studies have focused on CTCs in pediatric CNS embryonal tumors especially ATRT. This study aims to evaluate and compare the feasibility of detecting CTCs in young children with ATRT, and to analyze the clinical value of CTCs count in monitoring ATRT tumor progression.

**Methods:**

Young children under 3 years old who were diagnosed with ATRT and performed maintenance treatment from July 2023 to June 2024 after comprehensive therapy in our institution were enrolled. CTCs count both in cerebrospinal fluid (CSF) and peripheral blood were separately calculated based on two morphological types: CTC and tumor-derived circulating hybrid cells (CHC). Area under the receiver operating characteristic (ROC) curves (AUC) were used to determine the threshold of CTCs in predicting tumor progression. Kappa coefficients were applied to assess consistency between MRI scans, CSF cytology and CTCs by using progressive disease (PD) outcomes as the reference.

**Results:**

CTCs count in 34 blood samples and 34 CSF samples, as well as the results of CSF cytology examination and MRI scans in simultaneous period, were collected from six pediatric patients. When the progressive disease (PD) outcomes were used as a reference, CSF cytology test had a higher false negative rate compared with MRI scans (37.5% vs. 8.3%). In CSF, the sum of CTC + CHC had the highest significant diagnostic efficacy (AUC = 0.771, *p* = 0.001, Accuracy = 73.5%) with a cut-off value of 2.5. All of CSF-CTCs had statistically significant consistency with PD outcomes. In peripheral blood, all of CTCs had insignificant diagnostic efficacy. However, the sum of CTC + CHC had statistically significant consistency with PD outcomes (Kappa value = 0.406, *p* = 0.024), with a negative prediction cut-off value of 65 (AUC = 0.397, *p* = 0.371, Accuracy = 76.5%).

**Conclusion:**

CTCs in CSF and peripheral blood can both be detected in young-age ATRT patients after receiving comprehensive treatment. CTCs have considerable clinical predictive value in monitoring the progression of ATRT.

## Introduction

Atypical teratoid rhabdoid tumor (ATRT) is a rare and highly malignant tumor of the central nervous system (CNS), primarily occurring in young children under 3 years old [1]. The current mainstream treatment strategy typically includes a multi-modal approach involving surgical resection, high-dose chemotherapy and radiotherapy [2, 3]. Most of evidences suggest that early radiotherapy may be beneficial in ATRT treatment, however, due to the relatively high risk of long-term neurocognitive impairment after radiotherapy, craniospinal irradiation has been historically advised exclusively for pediatric patients above the age of 3 and for those exhibiting CNS dissemination at the time of diagnosis [4, 5], the treatment mainly consists of surgery and high-dose chemotherapy with autologous peripheral blood stem cell rescue to defer radiation as much as possible until the brain develops more maturely. Focal radiation is more acceptable and also be offered to children as young as 6 months old in some protocols, but the prognosis remains extremely poor [2, 6]. Early prediction of metastasis and comprehensive, macro-level intervention represent key of future directions in malignant tumors therapy [7]. Circulating tumor cells (CTCs) refer to tumor cells that detach from the tumor lesion and enter the peripheral blood circulation, playing a significant role in the process of tumor metastasis [8]. Previous research indicated that CTCs count hold significant potential value in predicting prognosis and formulating treatment plans for various solid tumors, as well as brain tumors [9–13]. However, there have been limited studies on CTCs in pediatric CNS tumors, particularly with regard to ATRT-specific CTC research. The aims of present study are to evaluate the feasibility of detecting CTC in both cerebrospinal fluid (CSF) and peripheral blood samples collected from children undergoing routine treatment for ATRT, additionally to analyze the clinical predictive value of CTCs count in monitoring tumor progression following ATRT comprehensive treatment in pediatric patients.

## Materials and methods

### Ethics approval

The present study conforms to the latest Declaration of Helsinki. All procedures including Magnetic resonance imaging(MRI) scan, CSF and blood collection were part of routine clinical examination items in processes of diagnosis, comprehensive treatment of CNS tumors and follow-up. Written informed consents were provided and signed by legal guardians of every pediatric patient. Data processing was pseudonymous and was approved by the local ethics board (registration number 202301302).

### Data collection and progression assessment

Data were collected retrospectively from patients in our institution under 3 years old, who were histologically diagnosed with ATRT and performed comprehensive treatment including operation resection, high-dose chemotherapy with the protocol of Head Start Ⅲ and maintenance treatment from July 2023 to June 2024. The inclusion criteria were the availability of collecting CSF and peripheral blood samples for the series of CTC test reports, and the children being determined to have tumor progression during the same period. The protocol of maintenance treatment was 12 cycles with 28 days in each cycle, with oral administration of tamoxifen 100mg/m^2^/dose (once daily, day 1–28) and ISOtretinoin 80 mg/m^2^/dose (twice daily, day 15–28), as well as intrathecal topotecan on the first day of each cycle (0.2 mg when age ≤ 1; 0.25 mg when age between 1 and 2; 0.32 mg when age between 2 and 3; 0.4 mg when age > 3). MRI scan was normally performed once per month in each patient during the chemotherapy and maintenance treatment for progression assessment. Each MRI scan was read and reported by a reporter and a reviewer from the neuroimaging specialist group. CSF cytology examination was applied before intrathecal topotecan injection routinely. Intrathecal topotecan would enhanced to twice weekly when CSF cytology was positive. Multidisciplinary specialists determined tumor progression based on the response definitions in the recommendations (response assessment in medulloblastoma and leptomeningeal seeding tumors) from Response Assessment in Pediatric Neuro-Oncology (RAPNO) committee [14].

### Quantify of circulating tumor cells

**Fig. 1:**
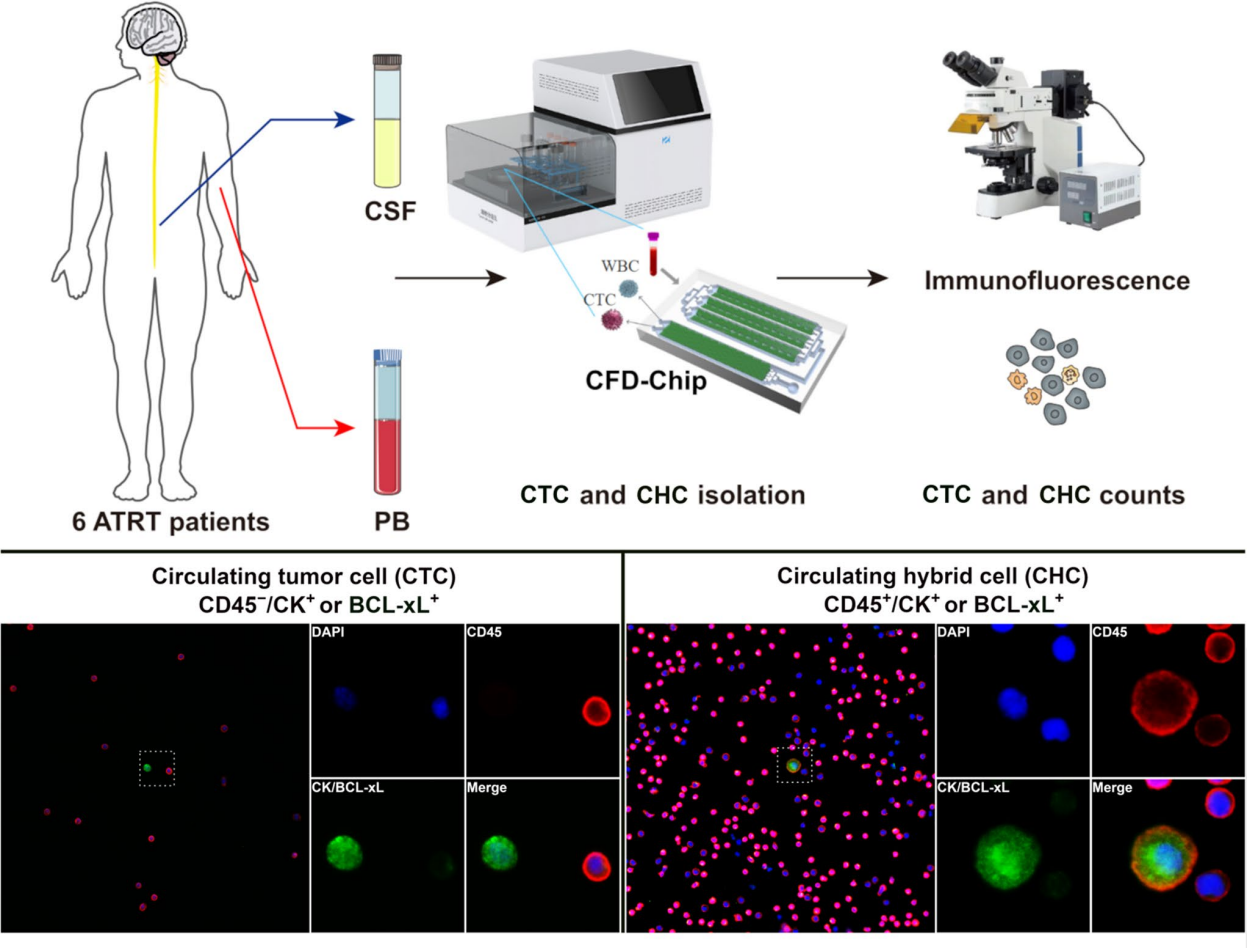
Circulating tumor cells (CTCs) would be separated and enriched by cascaded filter deterministic lateral displacement microchips (CFD-Chip) and integrated microfluidic system from cerebrospinal fluid (CSF) and peripheral blood (PB) samples. Different morphological types of CTCs including CTC and tumor-derived hybrid cells (CHC) were demonstrated after staining of DAPI (blue), CD45 (red) and CK/BCL-xL (green)

At each time point, four milliliter CSF sample and four milliliter peripheral blood sample were collected respectively for quantification of CTCs on the same day of CSF cytology examination. Samples were transported at low temperature to the sponsor’s laboratory immediately (normally within 6 h). The clinical status and private information of all patients were blinded to the operators, reporters and reviewers. In the present study, an integrated microfluidic system and cascaded filter deterministic lateral displacement microchips (CFD-Chip, Zigzag Biotechnology, Shenzhen, China) were utilized to enrich CTCs from samples diluted by four milliliters phosphate buffered solution (PBS) [15–18]. The enrichment process was completed in 25 min. The obtained cell suspensions were centrifuged at 250 g for 10 min and resuspended in PBS. The captured cells were profiled with immunofluorescence reagents and then identified by fluorescence microscopy. The expression of either Cytokeratins (CK) or B-cell lymphoma-extra large (BCL-xL) markers was identified as tumor cells. CD45 serves as a specific marker for white blood cells, while DAPI binds to the DNA of chromosomes and is commonly utilized for nuclear staining (Fig. 1). According to different staining results, CTC cells were classified into two morphological types:

A: CTC, which means the purely tumor cells in CSF or peripheral blood samples with CD45-/CK + or BCL-xL+/DAPI+;

B: CHC, which means circulating hybrid cells with CD45+/CK + or BCL-xL+/DAPI+.

### Statistical analysis

Receiver operating characteristic (ROC) curves were drawn to calculate the threshold of CTCs in tumor progression and evaluate clinical predictive value of CTCs. Kappa coefficients were calculated among MRI scans, CSF cytology examination, CTCs and the final determination results of tumor progression to evaluate the ability of various detection in assessing and predicting the progression of ATRT. The statistical significance was set at a p-value < 0.05.

## Results

### General information

**Fig. 2:**
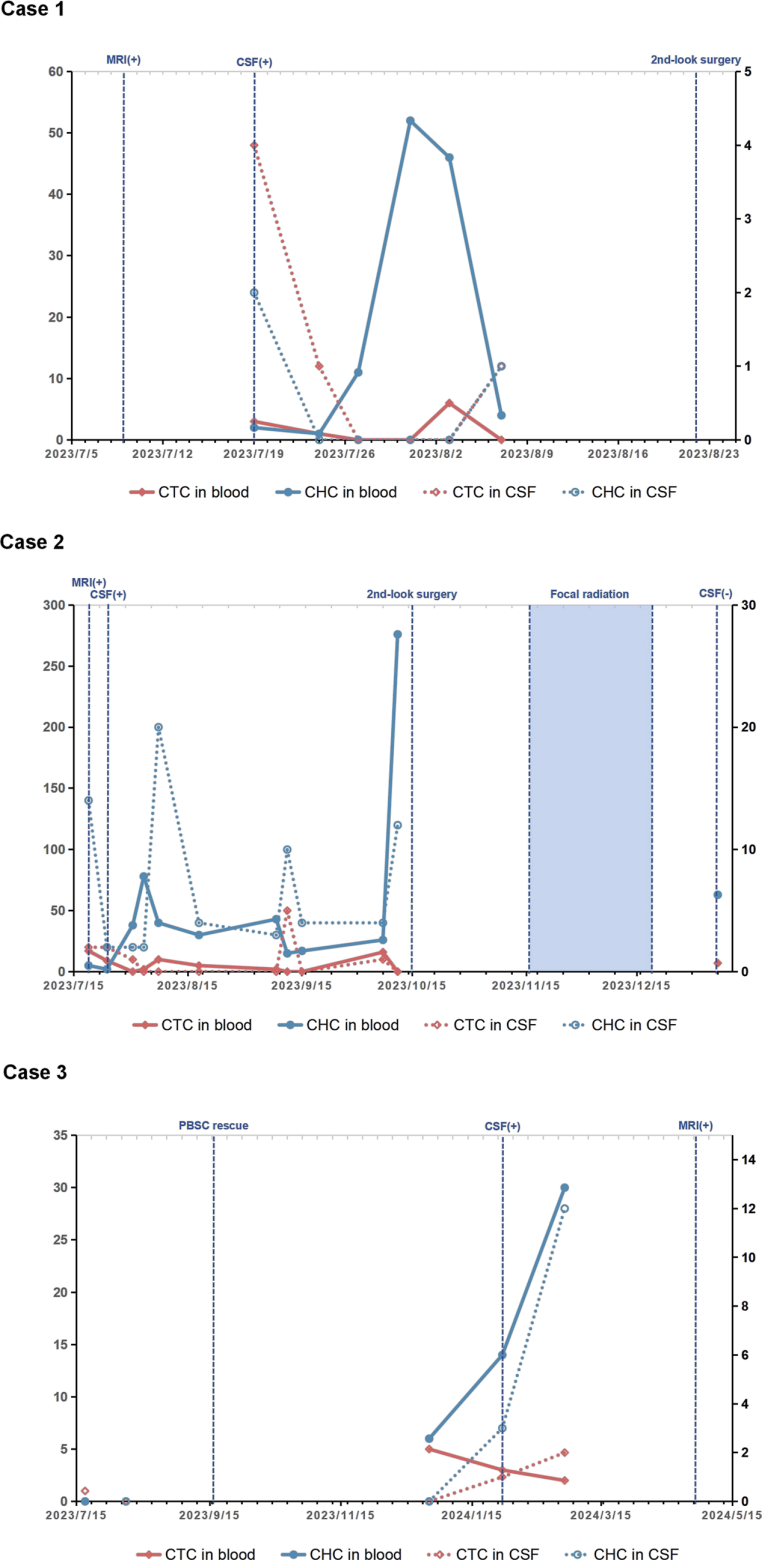

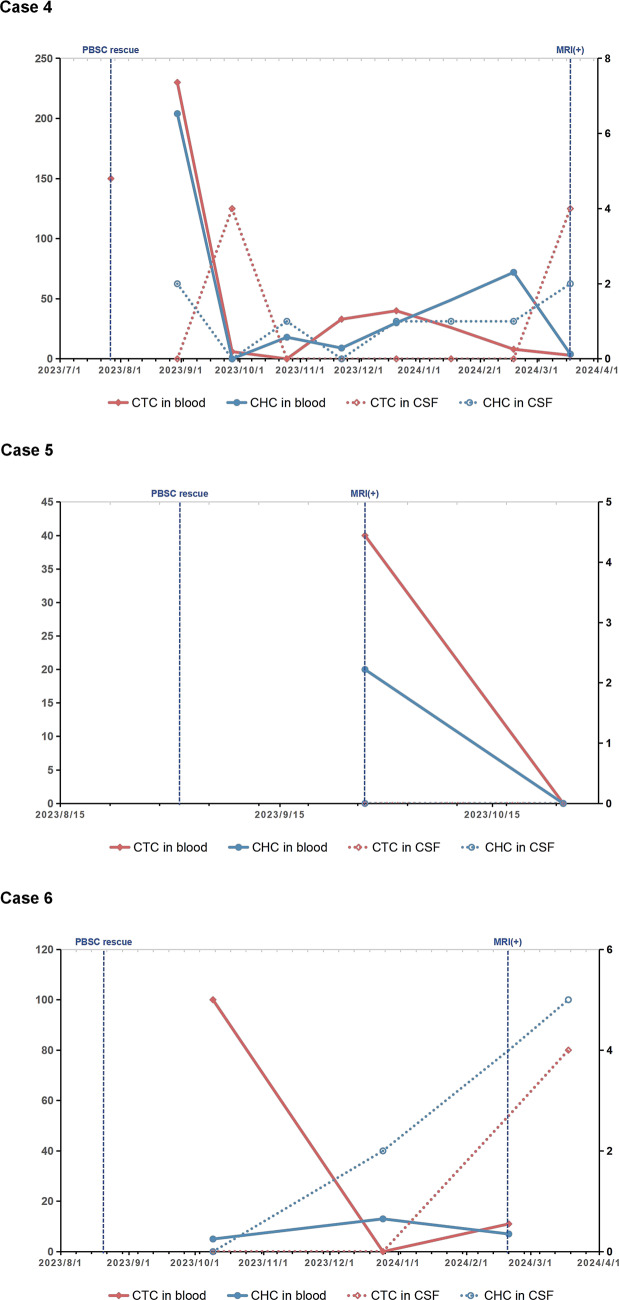
The figure illustrates the time points of circulating tumor cells (CTC) detection and treatment events before and after sampling for six pediatric patients. Among them, peripheral blood stem cells(PBSC) rescue represents the end point of high-dose chemotherapy, and cerebrospinal fluid cytology positive (CSF+) or MRI scan positive (MRI+) is marked as the time point of progressive disease. The results of CSF cytology examination were continuously negative in case 4, case 5 and case 6

A total of 37 CSF cytology tests and 28 MRI scans on six patients were involved in present study, with effective CTCs reports from 34 peripheral blood samples and 34 CSF samples (Fig. 2). General information of six pediatric patients including age at diagnosis, numbers of sample for CTCs detection, progression-free survival (PFS) after chemotherapy treatment and overall survival after diagnosis were illustrated in Table 1. The initial recurrence patterns that were determined to be progressive are also listed as progression pattern in the table. Among them, case 1 and 2 were later found to have CSF dissemination, while CSF cytology examination were continuously negative in case 4, 5 and 6. Three cases (case 1, 2 and 6) were considered as suspected progressive disease based on MRI scan before the samples collected and were confirmed by the following MRI scans and CSF cytology examinations. Two case (case 1 and 2) showed tumor progression before the start of consolidation chemotherapy, so high-dose chemotherapy was terminated and the 2nd-look surgery was arranged. The samples of these two cases were collected during the period from the termination of high-dose chemotherapy to the 2nd-look surgery, during which maintenance treatment protocol was used. The samples of the other four cases (case 3, 4, 5, and 6) were collected during the maintenance treatment stage after completing high-dose chemotherapy. Except for case 3, in which heterozygous mutation of SMARCA4 were detected in the blood samples of father and child, no hereditary cancer predisposition were detected in the other five cases. There is no metastatic lesion or CSF dissemination at diagnosis in all cases. Cases with local relapse (case 1, 2, 4, and 5) were received focal radiotherapy.

**Table 1:** General information of patients

No.	Gender	Age at diagnosis (months)	CSF	PB	2nd-look surgery after induction chemotherapy (Y/*N*)	PFS after chemotherapy (Days)	Progression pattern	Focal radiation (Y/*N*)	OS after diagnosis (months)
1	M	14	6	6	Y	0	Local relapse	Y	19.4
2	F	19	11	12	Y	0	Local relapse	Y	28.1*
3	M	19	4	4	N	134	CSF dissemination	N	29.0*
4	M	21	8	7	N	235	Local relapse	Y	41.0*
5	F	30	2	2	N	27	Local relapse	Y	31.1*
6	M	11	3	3	N	195	leptomeningeal dissemination	N	26.7*

*Follow-up till to 2025/06/21

CSF: cerebrospinal fluid; PB: peripheral blood; PFS: progression-free survival; OS: overall survival

### Consistency analysis of conventional examinations for ATRT progression

All 37 CSF cytology tests results and corresponding MRI results during the same period were compared by Kappa consistency test. Interval between two negative MRIs was regarded as a period of continuous negativity, and the converse held true. In three of six patients, CSF cytology remained negative constantly even after relapse lesions detected by MRI scan. Kappa coefficient between CSF cytology and MRI scans was 0.426 (*p* = 0.007), with a true positive rate of 59.1% and a true negative rate of 86.7% when taking MRI scans as the reference. When the results of progressive disease (PD) outcomes were employed as a reference, MRI scans demonstrated a higher degree of consistency compared with CSF cytology (Kappa coefficient: 0.885 vs. 0.539), with better sensitivity (91.7% vs. 62.5%). CSF cytology had a higher false negative rate compared with MRI scans (37.5% vs. 8.3%) (Tables 2 and 3).

**Table 2:** Kappa consistency analysis of MRI and CSF cytology

CSF cytology MRI	Positive	Negative	Kappa	*p* value*
Positive	13	9	0.426	0.007
Negative	2	13		

*Exact significance

**Table 3:** Kappa consistency analysis of progressive disease with MRI and CSF cytology

No. of cases		Progressive disease (PD)			
		Positive	Negative	Kappa	p value*
CSF cytology	Positive	15	0	0.539	< 0.001
	Negative	9	13		
MRI scans	Positive	22	0	0.885	< 0.001
	Negative	2	13		

*Exact significance

CSF cytology: Sensitivity = 62.5%, Specificity = 100%, Accuracy = 75.7%; MRI scans: Sensitivity = 91.7%, Specificity = 100%, Accuracy = 94.6%

### Predictive potential of CTCs in CSF

**Fig. 3:**
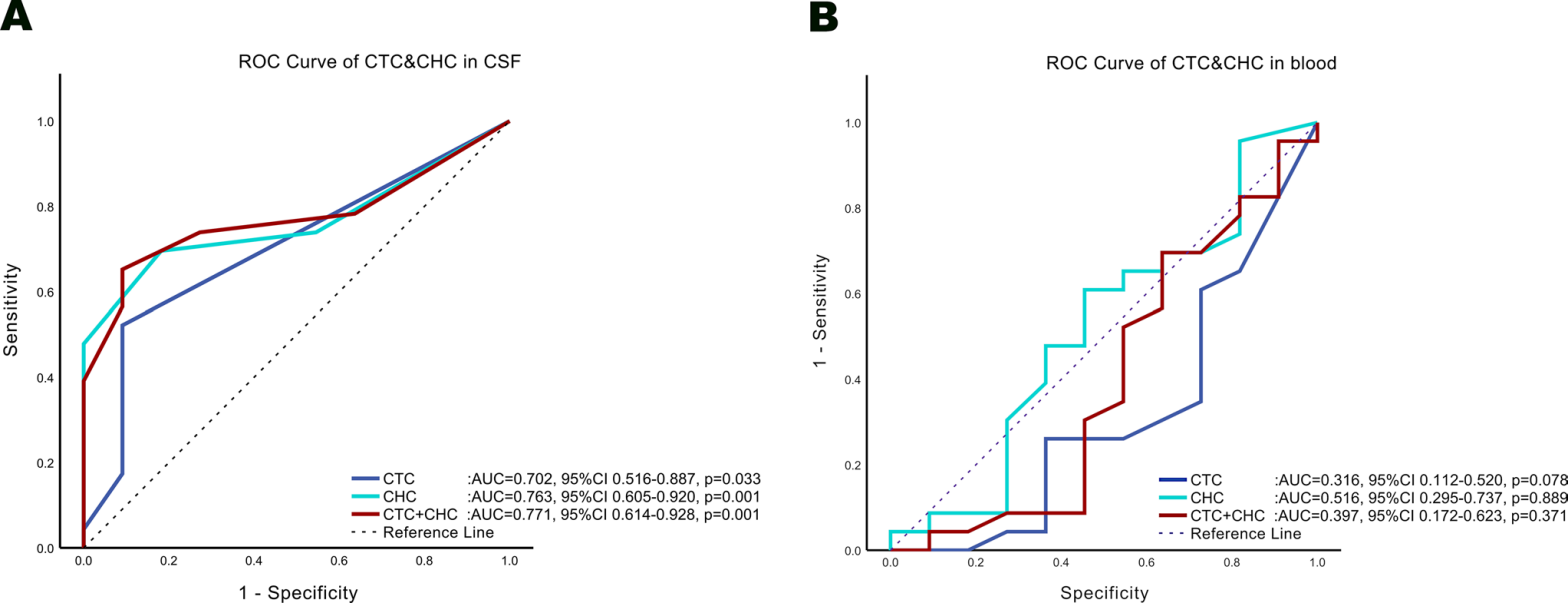
The figure shows the area under receiver operating characteristic (ROC) curves (AUC) of CTC, CHC, CTC + CHC in CSF (**A**) and in peripheral blood (**B**) separately. In CSF, the largest AUC of CTC + CHC indicate the most positive predictive performance. In peripheral blood, the smallest AUC of CTC indicate the most negative predictive performance

To evaluate the predictive potential of CTCs in CSF, area under ROC curves (AUC) of two CTC types based on the results of 34 CSF samples were calculated respectively. When the PD outcomes were used as a reference, all the AUC of CTC (0.702, *p* = 0.033), CHC (0.763, *p* = 0.001), and CTC + CHC (0.771, *p* = 0.001) were higher than 0.7 with significant diagnostic efficacy (Fig. 3A). The cut-off values in CTC, CHC and CTC + CHC were 0.5 (Sensitivity = 52.2%, Specificity = 90.9%, Youden’s Index = 0.431), 1.5 (Sensitivity = 69.6%, Specificity = 81.8%, Youden’s Index = 0.514) and 2.5 (Sensitivity = 65.2%, Specificity = 90.9%, Youden’s Index = 0.561), respectively. Due to the small sample size of present study, dividing it into an effective test set and validation set is not possible. Therefore, Leave-One-Out Cross-Validation (LOOCV) was utilized to verify the cut-off value and generalization performance of CSF-CTCs’ predictive ability. The average AUC and standard deviation of CTC, CHC and CTC + CHC after LOOCV were 0.702 ± 0.014 (Accuracy = 64.7%), 0.763 ± 0.014 (Accuracy = 73.5%) and 0.771 ± 0.014 (Accuracy = 73.5%), with the same cut-off values mentioned above in all 34 validations. The Kappa consistency test of CSF-CTCs with CSF cytology, MRI scans and PD outcomes was presented in Table 4.

**Table 4:** Kappa consistency analysis of CSF-CTCs with MRI, CSF cytology and progressive disease(PD)

CTCs in CSF (cut-off value)		CSF cytology				MRI				PD^#^			
		Positive	Negative	Kappa	*p* value*	Positive	Negative	Kappa	*p* value*	Positive	Negative	Kappa	*p* value*
CTC (0.5)	Positive	9	4	0.395	0.034	10	3	0.220	0.276	12	1	0.348	0.024
	Negative	6	15			11	10			11	10		
CHC (1.5)	Positive	11	7	0.357	0.045	14	4	0.344	0.076	16	2	0.459	0.009
	Negative	4	12			7	9			7	9		
CTC + CHC (2.5)	Positive	10	6	0.348	0.082	13	3	0.362	0.039	15	1	0.481	0.003
	Negative	5	13			8	10			8	10		

*Exact significance

^#^CTC: Sensitivity = 52.2%, Specificity = 90.9%, Accuracy = 64.7%; CHC: Sensitivity = 69.6%, Specificity = 81.8%, Accuracy = 73.5%; CTC + CHC: Sensitivity = 65.2%, Specificity = 90.9%, Accuracy = 73.5%

### Predictive potential of CTCs in blood

AUC of all CTC types based on 34 blood samples were calculated respectively. When using PD outcomes as a reference, the AUC of CTC (0.316, *p* = 0.078) and CTC + CHC (0.397, *p* = 0.371) were below 0.4 but with insignificant diagnostic efficacy (Fig. 3B). The negative prediction cut-off values in CTC, and CTC + CHC were 4 (Sensitivity = 65.2%, Specificity = 72.7%, Youden’s Index = 0.379) and 65 (Sensitivity = 91.3%, Specificity = 45.5%, Youden’s Index = 0.368). The average AUC and standard deviation of CTC after LOOCV were 0.316 ± 0.019 (Accuracy = 67.6%) with different CTC cut-off value of 5.5 in one of 34 validations. The average AUC and standard deviation of CTC + CHC after LOOCV were 0.397 ± 0.022 (Accuracy = 76.5%) with different CTC + CHC cut-off value of 61 in one of 34 validations. However, the AUC of CHC (0.516, *p* = 0.889) was around 0.5 with insignificant diagnostic efficacy and an unstable negative prediction cut-off value of 57.5 (Sensitivity = 91.3%, Specificity = 27.3%, Youden’s Index = 0.186). The average AUC and standard deviation of CHC after LOOCV was 0.516 ± 0.021, with a positive prediction cut-off value of 13.5 in three of 34 validations. The Kappa consistency test of peripheral blood-CTCs with CSF cytology, MRI scans and PD outcomes was presented in Table 5.

**Table 5:** Kappa consistency analysis of blood-CTCs with MRI, CSF cytology and progressive disease(PD)

CTCs in blood (cut-off value)		CSF cytology				MRI				PD^#^			
		Positive	Negative	Kappa	*p* value*	Positive	Negative	Kappa	*p* value*	Positive	Negative	Kappa	*p* value*
CTC (4)	Positive	11	7	0.357	0.045	13	5	0.225	0.291	15	3	0.339	0.066
	Negative	4	12			8	8			8	8		
CHC (57.5)	Positive	13	16	0.022	1.000	19	10	0.154	0.348	21	8	0.217	0.300
	Negative	2	3			2	3			2	3		
CTC + CHC (65)	Positive	13	14	0.120	0.426	19	8	0.317	0.079	21	6	0.406	0.024
	Negative	2	5			2	5			2	5		

*Exact significance

^#^CTC: Sensitivity = 65.2%, Specificity = 72.7%, Accuracy = 67.6%; CHC: Sensitivity = 91.3%, Specificity = 27.3%, Accuracy = 70.6%; CTC + CHC: Sensitivity = 91.3%, Specificity = 45.5%, Accuracy = 76.5%

## Discussion

The current study shows that in ATRT patients with young age who are treated after operation and high-dose chemotherapy, neither MRI scan nor CSF cytology can completely eliminate the occurrence of false negatives with a single test in detecting tumor progression or relapse. CSF cytology examination has a higher false negative rate, thus resulting in lower sensitivity. This preliminary study with small size data demonstrated the predictive potential of CSF-CTCs in ATRT tumor progression with acceptable diagnostic consistency, competitive accuracy and lower false negative rate compared with CSF cytology. Furthermore, the detection of total number of CTC + CHC cells has the highest significant predictive ability with the largest AUC among the CSF-CTCs. The predictive potential of peripheral blood-CTCs with a certain degree of accuracy and impressive sensitivity (91.3%) and significant consistency with PD outcomes, however, is limited in detecting ATRT tumor progression with excessively high false positive rates (54.5%) in present study.

CSF cytology examination has been mentioned in several existing protocols for ATRT treatment and is one of the important bases for assessing tumor staging and formulating treatment plans [2, 6, 19]. CSF cytology has been reported with a relatively low sensitivity of 44–67% but with a high specificity of 100% [20, 21]. Our study observed that CSF cytology with a relatively high false negative rate of 37.5% in children with ATRT after treatment, which was consistent with previous studies, suggesting that a single negative CSF cytology result should be suspected. Multiple tests or combined with other detection methods are needed to improve accuracy and promptly identify CSF dissemination [20].

In terms of liquid biopsy of CNS tumors in clinical applications, CSF-CTCs analysis has been mostly applied as a diagnostic tool of leptomeningeal metastases from extra-CNS tumors with significant added prognostic value [13, 21]. Several studies have applied CSF-CTCs detection in primary CNS high-grade glioma [10, 22]. CSF-CTCs analysis offers advantages over CSF cytology, including increased sensitivity, objectivity, and ability to quantify disease burden [21, 23]. Due to the relative ease of obtaining blood samples compared to CSF, peripheral blood-CTCs analysis in primary CNS tumors has been more extensively researched in previous studies [10–12, 22, 24, 25]. Zhang et al. summarized the previous studies on blood-CTCs related to glioma, all of which had high sensitivity in conclusion but still had shortcomings including low detection rate, insufficient sample size, and high false positive rate [11]. Juan Y-C et al. collected blood and CSF samples from six patients with glioblastoma to perform dynamic monitoring of CTCs in relation to disease progression, which observed a higher number of CTCs in CSF compared to blood, and the levels of CTCs in both body fluids were found to vary with tumor progression, with a negative correlation between CSF-CTCs and blood-CTCs, suggesting that CTCs in these two body fluids may reflect different aspects of the disease biology [22]. Garzia et al. provided evidence that medulloblastoma CTCs in the blood of therapy-naive patients, as well as in vivo through flank xenografting and parabiosis, can disseminate through the bloodstream to the leptomeningeal surface of the brain and spinal cord, resulting in leptomeningeal metastases [12]. The current study provides evidence supporting the potential clinical value of detecting CSF-CTCs in young children with ATRT for monitoring tumor progression, allowing for earlier determination of whether the tumor has progressed, and enabling more timely salvage treatment. In our study, CTCs presented a relatively high false positive rate in blood samples when traditional detection methods were used as the reference, however, the phenomenon that CTCs can still be detected both in blood and CSF in young children suffering with ATRT after comprehensive treatment cannot be ignored. The crossover of trend lines of CSF-CTCs and blood-CTCs, as well as the negative prediction potential of CTCs in peripheral blood, can be clearly observed, which also indicates that the peaks of CTCs in the two different body fluids may occur at different stages of tumor progression.

Currently, CTC detection techniques are divided into two major approaches: biomarker-dependent biological sorting methods, and biomarker-independent physical separation methods. Biological sorting methods enrich CTCs by positive selection using CTC capture antibodies, or negative selection by removing white blood cells, macrophages, and platelets with CD45 and CD61 [26]. However, no universal tumor cell biomarker currently exists, making it challenging to completely avoid the omission of CTCs. Physical separation methods refer to the separation and enrichment of CTCs from other cells based on their physical characteristics such as size, density, mechanical and electrical properties, etc. The microfluidic chip method enriches CTCs by taking advantage of the differences in hydrodynamic properties such as deformability of different cells under specific conditions [26]. The advantage of physical separation lies in its ability to facilitate the detection of CTCs that express heterogeneous antigens or CTC subtypes that express unknown antigens. The cascaded filter deterministic lateral displacement microchips mentioned in present study have been reported and recently employed for CTCs enrichment of neuroblastoma successfully [18]. However, it may also have disadvantages including low separation purity and the possibility of missing a few smaller tumor cells. Due to the immunohistochemical diversity of ATRT [27], the biological sorting method may lead to a relatively high rate of missed detection of ATRT-CTCs. Physical enrichment methods such like microfluidic chips may be more practical for the enrichment of ATRT-CTCs. To the best of our knowledge, there is currently no unified standard for immunohistochemical markers specifically to detect ATRT-CTCs. I. Batth et al. reported previously that cell surface vimentin was utilized to conduct CTC detection on the blood samples of nine children including one ATRT patient, only seven had CTC detected in their blood, without explicitly mentioning that the ATRT patient was among these seven children [28]. In present study, CK and BCL-xL were employed for the immunofluorescence detection of ATRT-CTCs. ATRTs are a heterogeneous tumor type and rhabdoid cells are a defining characteristic of ATRTs. Epithelial markers of CK can be expressed in rhabdoid cells, reflecting their variable epithelial differentiation. Most ATRTs express proteins such as CK that originate from epithelial cells, which was reported in previous studies [27, 29]. BCL plays a crucial role in the proliferation and division of ATRT, BCL-2 inhibitors have significant effects on different tumor cell clusters in different ATRT subtypes [30]. The detection of ATRT-CTCs by BCL-xL marker, which also belongs to the BCL-2 family and plays a crucial role in regulating the intrinsic pathway of apoptosis, suggests that CTC detection has the potential to monitor the recurrence of tumor subclones and to guide drug screening.

Previous clinical studies have rarely mentioned the predictive value of different forms of CTCs in tumor progression. The majority of CTCs exist in a single-cell state, certain CTCs travel as multicellular clusters, which have been shown to confer survival advantages and exhibit enhanced metastatic potential [31]. Nevertheless, the interaction between CTCs and the immune system is a critical point by which CTCs facilitate metastatic progression [32]. During the progression of brain tumors, the blood-brain barrier is damaged and leads to an increase in the permeability of the neurovascular unit [33]. Under the guidance of chemokines, immune cells from the peripheral blood could pass through the vascular endothelial cells to enter the CNS and could be detected in brain tumors [34]. On the other hand, the detection of CTC in the peripheral blood also indicates that during the progression of brain tumors, tumor cells can penetrate the blood-tumor barrier and enter the circulatory system. CTC can be fused with the leukocyte populations in the immune system to form tumor-derived hybrid cells (CHC) [32, 35]. As macrophages are the major leukocyte cells in tumor-derived hybrid cells, CHC express pan-leukocyte antigen CD45 and tumor markers [35]. Fused hybrid cells are more frequently observed in circulation compared to single CTC and maintain the full functional characteristics, including both genotypic and phenotypic properties of leukocytes and tumor cells [35]. Furthermore, as a favorable form for CTC to survive under the immune system and chemotherapy, CHC still retains tumorigenicity and correlates with disease stage and patient survival [35, 36]. We separately tallied the quantities of CTC and CHC both in blood and in CSF, and analyzed the predictive value of each form of CTCs for tumor progression. The results indicated that the quantity of CTC + CHC had a greater predictive value for tumor progression, providing a direction and idea for research in the future.

The limitations of this study lie in the small sample size. This study is a single-center study, thus selection bias exists. More clinical data are still needed for rare tumors like ATRT. The clinical predictive value of ATRT-CTCs detection in the progression of ATRT still requires more comparative studies. Additionally, a quantity analysis of ATRT-CTCs for treatment-naive cases, an investigation into the changes in ATRT-CTCs under various treatment modalities such as surgery, chemotherapy, radiotherapy and immunotherapy, as well as the research into the underlying mechanisms of ATRT-CTCs transfer between blood and CSF, are also needed for further research.

## Conclusion

Circulating tumor cells (CTCs) can be persistently detected in both cerebrospinal fluid (CSF) and peripheral blood obtained from young children diagnosed with atypical teratoid rhabdoid tumor (ATRT) following comprehensive treatment. The analysis of CTCs in CSF demonstrates potential for monitoring ATRT progression and exhibits predictive accuracy comparable to that of conventional diagnostic methods including CSF cytology and magnetic resonance imaging (MRI). While CTCs in peripheral blood shows the relatively high sensitivity, the high false positive rate constrains the clinical value in predicting tumor progression among pediatric ATRT patients.

## Data Availability

No datasets were generated or analysed during the current study.

## Abbreviations

ATRT: Atypical teratoid rhabdoid tumor
AUC: Area under the curve
BCL-xL: B-cell lymphoma-extra large
CHC: Circulating hybrid cells
CI: Confidence interval
CK: Cytokeratins
CNS: Central nervous system
CSF: Cerebrospinal fluid
CTC: Circulating tumor cells
LOOCV: Leave-One-Out Cross-Validation
MRI: Magnetic resonance imaging
PBSC: Peripheral blood stem cells
PD: Progressive disease
PFS: Progression-free survival
ROC: Receiver operating characteristic
